# Analysis of Advantages and Disadvantages of the Location Methods of International Auricular Acupuncture Points

**DOI:** 10.1155/2016/2806424

**Published:** 2016-03-31

**Authors:** Pei-Jing Rong, Jing-Jun Zhao, Lei Wang, Li-Qun Zhou

**Affiliations:** ^1^Institute of Acupuncture and Moxibustion, China Academy of Chinese Medical Sciences, Beijing 100700, China; ^2^School of Acupuncture-Moxibustion and Tuina, Beijing University of Chinese Medicine, Beijing 100029, China; ^3^School of Basic Medical Science, Beijing University of Chinese Medicine, Beijing 100029, China

## Abstract

The international standardization of auricular acupuncture points (AAPs) is an important basis for auricular therapy or auricular diagnosis and treatment. The study on the international standardization of AAPs has gone through a long process, in which the location method is one of the key research projects. There are different points of view in the field of AAPs among experts from different countries or regions. By only analyzing the nine representative location methods, this paper tried to offer a proper location method to locate AAPs. Through analysis of the pros and cons of each location method, the location method applied in the WFAS international standard of AAPs is thoroughly considered as an appropriate method. It is important to keep the right direction during developing an International Organization for Standardization (ISO) international standard of auricular acupuncture points and to improve the research quality of international standardization for AAPs.

## 1. Introduction

The third regional conference of the working group of the standardization of acupuncture points was held in Seoul, South Korea, in 1987 [1]. In this conference, the China Association of Acupuncture-Moxibustion put forward a draft of international standard of auricular acupuncture points (AAPs) including ninety points. The draft was seriously discussed and the board set up three criteria of selection: (1) points which use international and common names; (2) points whose therapeutic values are well proven; (3) points whose location in the auricular area appears to be generally well-defined. Forty-three points that fulfilled all three criteria were classified with an alphanumeric code (a two-letter abbreviation and one number), pinyin, Han character, and English name. Thirty-six points that fulfilled the first and second criteria were not attributed a code, but only pinyin, Han character, and English nomenclature. Eleven points that failed to fulfill all three of the criteria were excluded. After a last inconclusive 1990 WHO meeting [2] in Lyon, Chinese researchers continued the standardization work and mainly continued due to the contribution of the publication of Chinese National Standard in 1993 [3]. This document was updated in 2008 [4] by a second document.

An international standard of AAPs is an inevitable requirement when the auricular diagnosis and treatment medicine develops for a long period and goes to a high level. Through more than 30 years of effort, great progress and achievements have been made in researches on the international standard of AAPs. Up to now, an international standard has been formulated and issued by the World Federation of Acupuncture-Moxibustion Societies. An International Organization for Standardization (ISO) international standard is also in development. Nevertheless, there are still debates on the location methods for locating AAPs, including location based on “points,” “subzones,” “coordinate,” and other methods.

The corresponding author of this paper has been engaged in the study on the international standardization of AAPs since 1986 and had the honor to participate in the whole process of research work on the Chinese national standard of AAPs and international standard of AAPs. As the chief expert of the project, he finished the international standard for auricular acupuncture point [5], which was issued by the World Federation of Acupuncture-Moxibustion Societies. Therefore, he had a comprehensive understanding on the background, content, advantages, and disadvantages of different location methods during developing this international standard. This paper aimed to analyze these location methods and provide a reference for the formulation of an ISO standard of AAPs in the future.

## 2. Methods

From perspectives of historical context of AAPs, clinical inheritance, and practice of AAPs, the understanding of the essence of points, advantages, and disadvantages of the following nine location methods for locating AAPs were analyzed, including (1) the location method based on points for the anterior of the auricle, (2) the location method based on subzones for the anterior of the auricle, (3) the location method based on the divided subzones according to anatomy of the anterior of the auricle, (4) the location method based on Nogier's Point Zero for the anterior of the auricle, (5) the location method based on the facial ties of the knee of the helix and lobe, (6) the location method based on both points and subzones of the anterior of the auricle, (7) the location method based on smaller subzones of the anterior of the auricle, (8) the location method based on subzones, points, and lines for the anterior and posterior of the auricle, and (9) the location method based on subzones, points, and lines for full cover of the anterior and posterior of the auricle. The analysis is described in detail with pictures so that readers can understand easily.

## 3. Results and Discussion

### 3.1. The Location Method Based on Points for the Anterior of the Auricle

**Figure 1:**
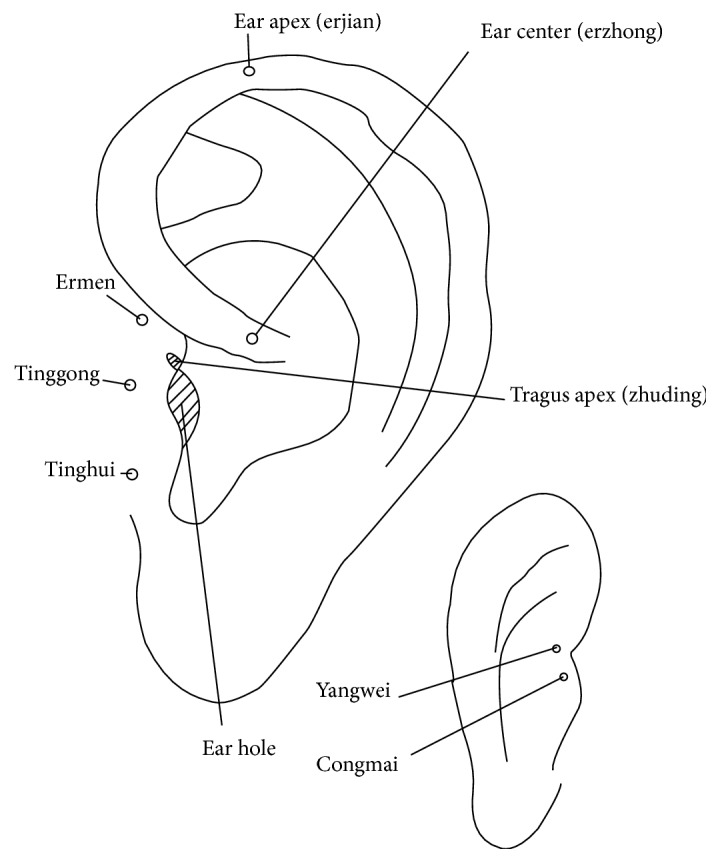
AAPs described by Chinese experts before the 18th century, recorded in Professor Huang Li-Chun's book, Auricular Diagnosis and Treatment.

**Figure 2:**
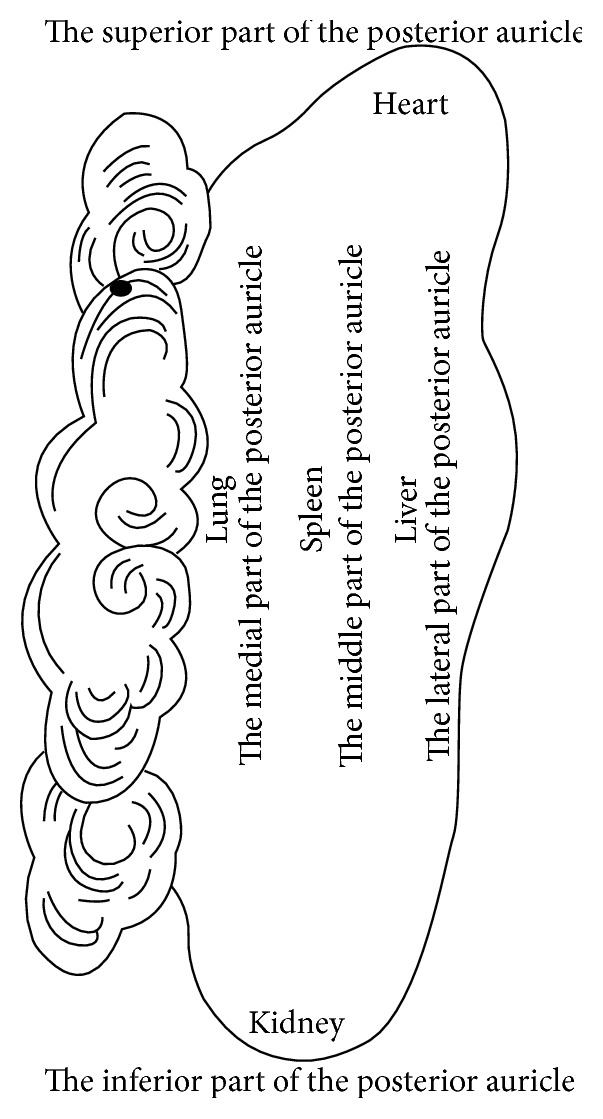
The posterior auricular map in 1888.

**Figure 3:**
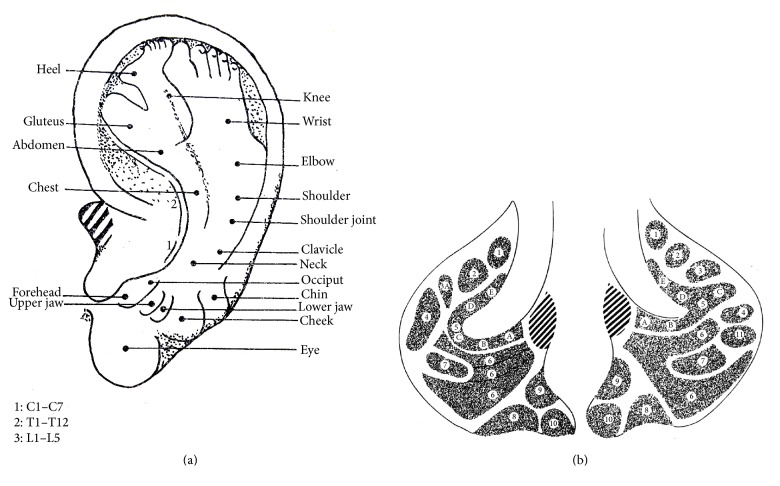
(a) Paul Nogier's findings of inverted fetus on the auricle representing the somatic structures in 1957. (b) Paul Nogier's findings of inverted fetus on the auricle representing viscera and organs in 1957. (1) Bladder; (2) kidney; (3) pancreas; (3A) gallbladder; (4) liver; (5A) oesophagus; (5B) cardia; (5C) stomach; (5D) small intestine; (5E) large intestine; (6) lung; (7) heart; (8) subcortex; (9) internal nose; (10) endocrine; (11) spleen.

No specific illustration of the location of AAPs can be found in Chinese ancient medical books [6] (Figure 1 [7]), except the posterior auricular map [8] (Figure 2) with the theory, where each of the five areas of the posterior of the auricle corresponded to each of the five Zang-organs, including heart, liver, spleen, lung, and kidney. Therefore, it is difficult to identify whether there was the thought of location of AAPs based on points at the old time. The ear reflex map of French physician, Paul Nogier, was introduced to China in 1958 by Dr. Ye Xiao-Lin. And this map was the earliest representative systematic auricular map in modern time [9–11] (Figure 3(a) [9] and Figure 3(b) [12]). The location method based on points and subzones is adopted in his two maps. The location method based on points is applied to represent the somatic structures of the body. Although the thought of location method of subzones to represent viscera and organs of the body was used, Figure 3(b) is only a sketch map. The location method based on points has three advantages. Firstly, the original is respected and it can comprehensively reflect the historic appearance of the development of auricular therapy in China and France. Secondly, it is visualized and it can effectively guide the clinical application of AAPs. Thirdly, those points which are marked at tips of the different zones of the auricle (e.g., the tip of the tragus) or notches (e.g., the intertragic notch) are easy to describe the location accurately with auricular anatomical terms. With the above advantages, this location method was adopted in the earliest draft of the international standard of AAPs. However, it has also some limitations. At first, it cannot accurately reflect the corresponding somatic structures, viscera, and organs reflexed on the auricle. Then, those points that are not located at the tips of the different zones of the auricle or notches cannot be accurately marked and described with auricular anatomical terms. Thirdly, it cannot cover the whole auricle. As a result, this location method was not the only location method in the later drafts.

Figure 2 is from Zhang Zhen-Jun's (Zhang Di-Shan's) book,* Essential Techniques for Massage*. The superior part of the posterior auricle corresponds to heart, the inferior part corresponds to kidney, the middle part corresponds to spleen, the lateral side corresponds to liver, and the medial side corresponds to lung.

### 3.2. The Location Method Based on Subzones for the Anterior of the Auricle

**Figure 4:**
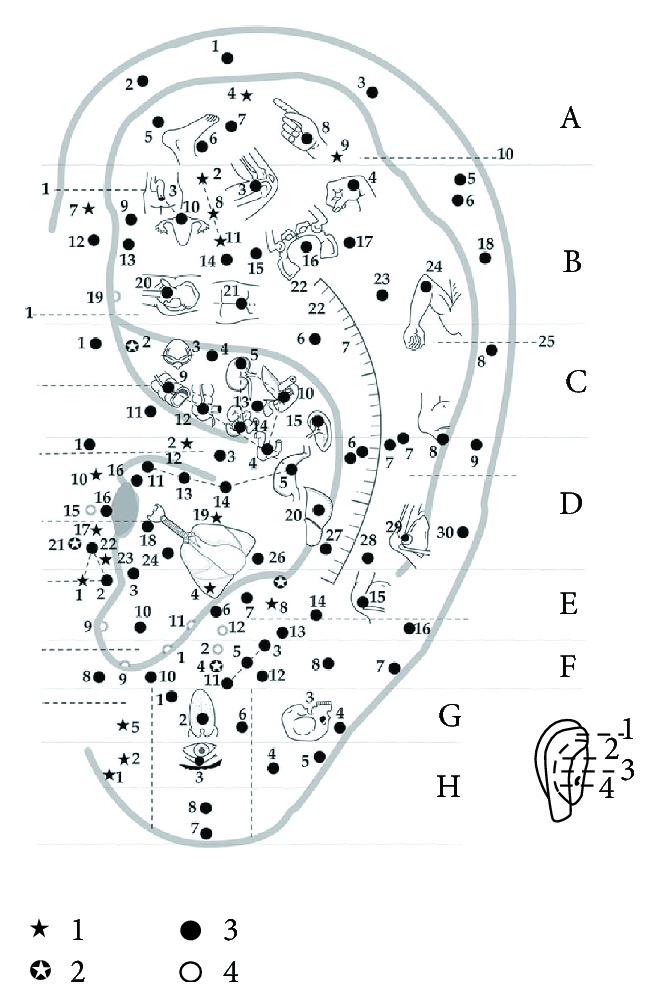
Dr. Jarricot's auricular map in 1973.

**Figure 5:**
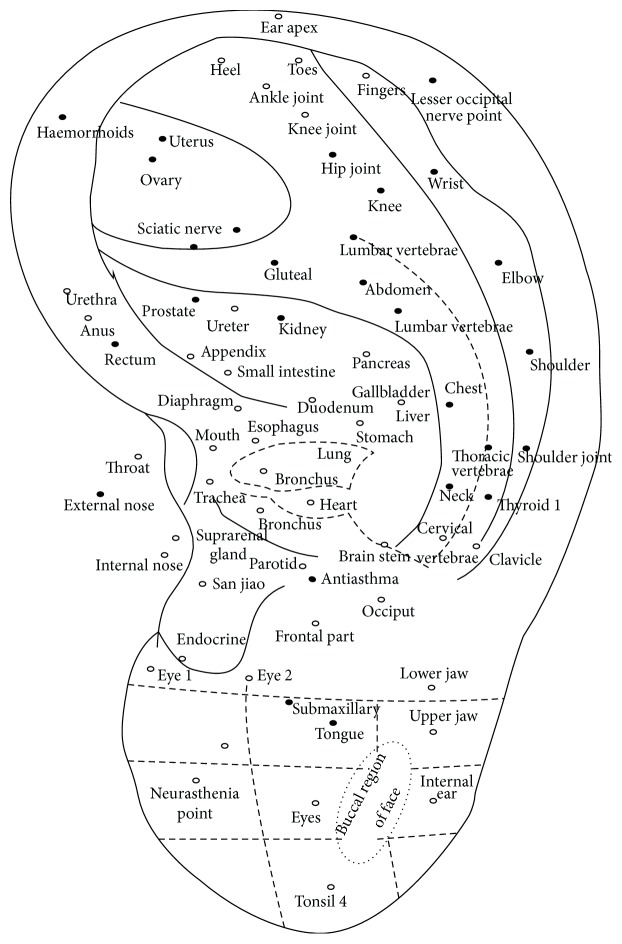
Dr. Wang Zhong's auricular map in 1984.

With awareness of the limitations of the location method based purely on points, the Chinese academic circle in the late 1980s tried to locate AAPs according to the subzones. There were different zones on the auricle according to the anatomical structures of the auricle. Each zone was further divided into different subzones. The work began with the aim to locate AAPs at the ear lobe and concha where it was difficult to use points to locate these AAPs. The subdivision in vertical and horizontal lines of the ear lobe was published by Jarricot and Wong [13], who divided the whole outer auricle in horizontal stripes ((A) to (H), from top to bottom) which was called “the Great Ear of the School of Chen-Yang” (Figure 4). A Chinese physician, Dr. Wang, made also the exploration to locate AAPs by subzones in his book [14] (Figure 5), Auricular Acupuncture. According to the Chinese history of acupuncture, auricular acupuncture, and Nogier's auricular maps introduced into China in 1958, the location method based only on points is used to name acupuncture points, or AAPs. As a breakthrough of the thinking inertia, auricular point is just a point, and this location method based on subzones not only walked a significant step forward from perspectives of the clinical application of AAPs and description of the location of AAPs, but also provided an innovative idea for future study on the location of AAPs. The shortcoming of this method is that the division of zones is not comprehensive and standardized and lacks anatomical basis. Most of the AAPs are located by the location method based on points. The location method based on subzones is only applied to locate AAPs on the lobe, the inferior concha. There is not a general principle to specify the subzones, which makes it difficult to duplicate on different people. There is no description of anatomical borders, which can divide the auricle into zones and subzones.

### 3.3. The Location Method Based on the Divided Subzones according to Anatomy of the Anterior of the Auricle

**Figure 6:**
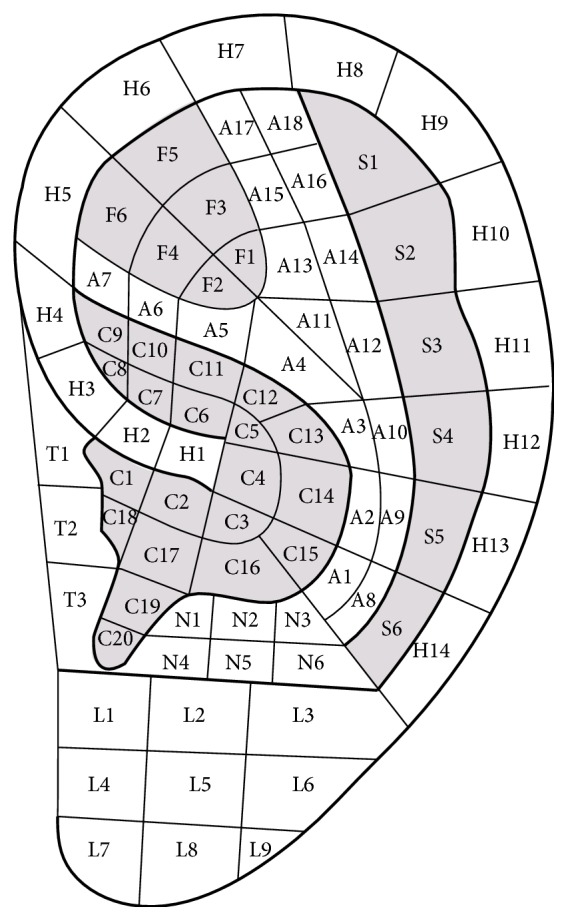
Oleson's nomenclature of subzones in 1983.

In 1990, Oleson and Kroening, scholars from University of California, Los Angeles, the United States of America, first located all AAPs at the visible surface of the anterior auricle according to the anatomy of the auricle in his article [15] (Figure 6). Each subzone was coded by the initials of its corresponding anatomical structure and a number. For example, the first subzone of triangular fossa is short for TF1 and the second subzone of triangular fossa is short for TF2. His original intention was to ease the dispute over the problem-one name with different positions, or one auricular acupuncture point (AAP) with different names in Chinese and French auricular acupuncture systems, and to advocate neglecting the traditional names of AAPs but to name them with codes. Innovations of this location method are as follows. Firstly, location of AAPs is completely based on subzones instead of points, breaking through the limitations of the location method based on points. Secondly, the location method based on the divided subzones according to anatomy of the auricle has been proved to be the right direction to locate AAPs. Thirdly, the coding system, the name of the anatomical structure and a number, is simple and clear. Fourthly, according to the theory of bioholographic law, AAPs are the projections of the body parts on the auricle and therefore the location method based on the divided subzones can objectively reflect the correspondence between AAPs and the body parts. The shortcomings are as follows. Firstly, tips of the different zones of the auricle or notches are not proper to be marked as subzones. Secondly, the academic value of the historical evolution and clinical significance of AAPs are neglected. Thirdly, those AAPs at the junction of the neighboring anatomical structures of the auricle cannot be straight forwardly and accurately marked. Fourthly, the border of the neighboring anatomical structures of the auricle, such as scapha and antihelix, cannot be clarified and lacks detailed anatomical evidence. Finally, the posterior of the auricle is not divided into zones and further subzones.

### 3.4. The Location Method Based on Nogier's Point Zero for the Anterior of the Auricle

**Figure 7:**
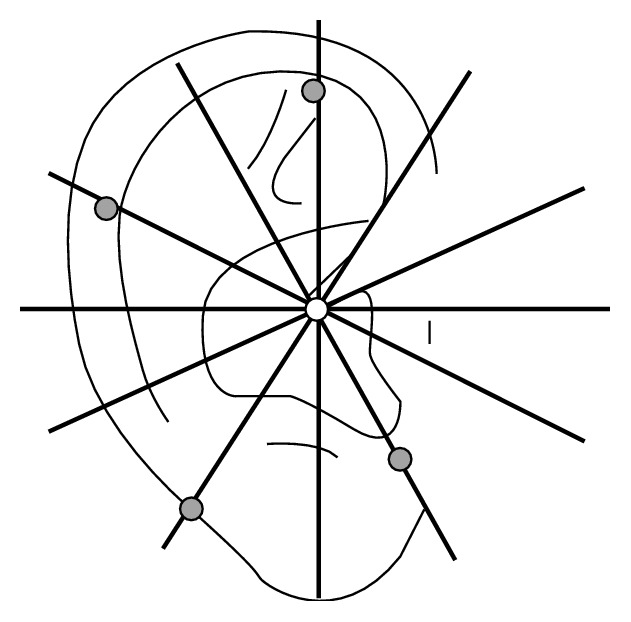
Dr. Rouxeville's location method of auricular acupuncture points in 1980.

**Figure 8:**
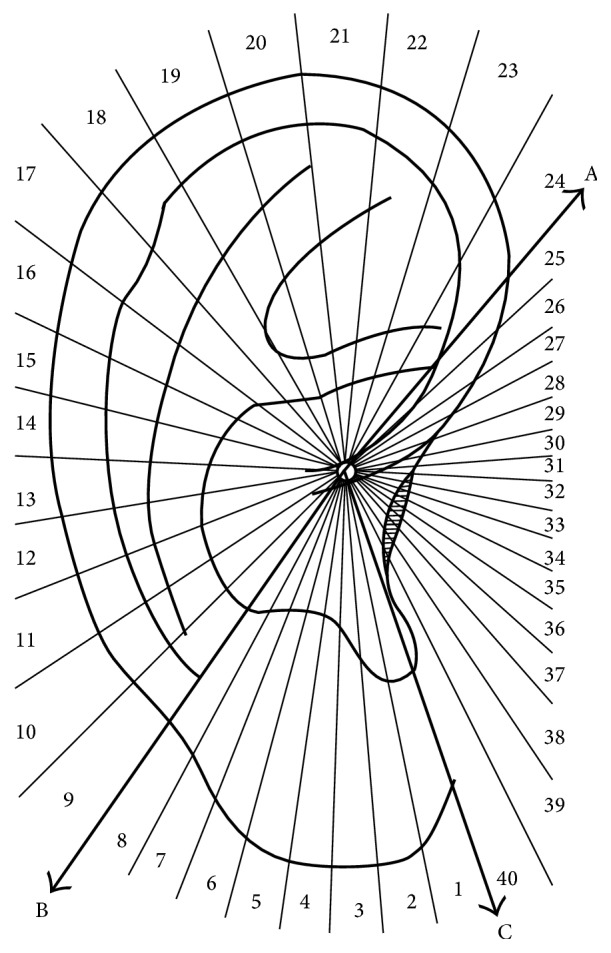
Professor Romoli's location method of auricular acupuncture points in 2009. Auricular sectogram, centered on Nogier's Point Zero, with the three half-lines (A), (B), and (C) subdividing the auricle.

This method was proposed in 1990 by Dr. Bossy at the international conference on AAPs held by the WHO Regional Office for the Western Pacific in Lyon, France, to solve the long-troubling problem of international standardization of AAPs. The zero point was set at the helix crus notch and the auricle is divided into four quadrants by the horizontal and vertical axis. AAPs were marked by the intersections of the latitude and longitude lines in each quadrant. Dr. Rouxeville [16] further divided each quadrant into three equal sectors (Figure 7). Three other authors, the Russian Durinyan [17] and Romoli and Mazzoni [18] from Italy, also developed a grid with a variable number of sectors centered in Point Zero. The sectogram was obtained by subdividing the auricle into three semiaxes (A), (B), and (C) going, respectively, through the visual intersection point of the posterior edge of the raising branch of the helix with the lower branch of the antihelix (A) and through the antitragus-antihelix groove (B) and tangent to the posterior edge of the tragus (C). The resulting main sectors (A)-(B), (A)-(C), and (B)-(C) were subdivided, respectively, into 16 sectors, (A)-(B) and (A)-(C), and into 8 sectors, (B)-(C) (Figure 8). The location method based on Nogier's Point Zero for the anterior of the auricle has the thought of locating AAPs with subzones. The advantage of this method is that it could accurately locate every point with no controversy about the nomenclature and locations of AAPs. However, as the saying goes, “no leaves are exactly the same in the world,” no ears have exactly the same shape. The latitude and longitude coordinates hardly match the actual positions of AAPs based on anatomical structures, which therefore is difficult to serve as a repeatable, standard location method. Up to now, this location method is not applied extensively enough.

### 3.5. The Location Method Based on the Facial Ties of the Knee of the Helix and Lobe

**Figure 9:**
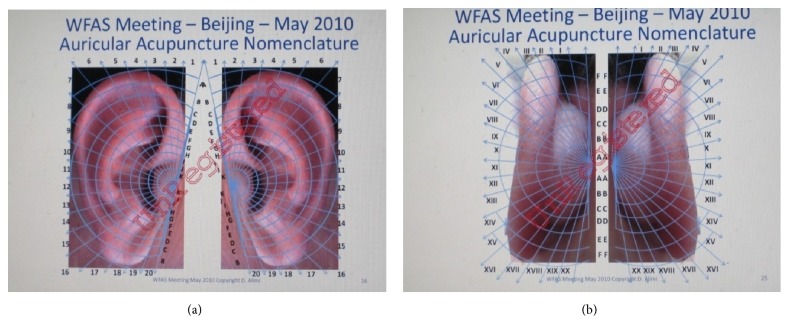
(a) Dr. Alimi's location method for the anterior of the auricle in 2010. (b) Dr. Alimi's location method for the posterior of the auricle in 2010.

This location method (Figures 9(a) and 9(b)), proposed by Dr. David Alimi, took for base the anatomical structures of the ear as the ties which were constant at all humans: namely, the facial ties of the knee of the helix and lobule. These ties (their epicenter) were always aligned on a perfect right which always passed by the corpus callosum [19]. The corpus callosum connects the left and right cerebral hemispheres and facilitates interhemispheric communication. The point was considered to represent the corpus callosum, called 0 premium by the largest number, pulled tangents in these ties. A semicircle of an angular value of 180 degrees was divided into 20 equal angles, with 9 degrees for each sector. The group was a segmentogram recovering the totality of the anterior and posterior surfaces of the auricle. This location method is based on the division of subzones. However, this location method is not based on the anatomical structures of the auricle, and it is more complicated than Oleson's location method in 1983 and Chinese location method based on points and subzones when applied in clinical practice [20].

### 3.6. The Location Method Based on Both Points and Subzones of the Anterior of the Auricle

**Figure 10:**
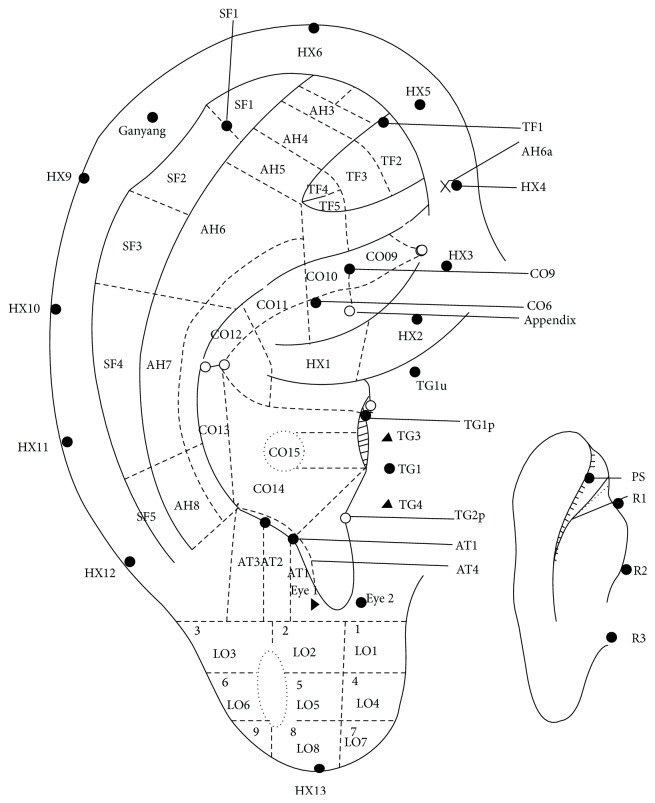
Standard project of auricular acupuncture points by the China Association of Acupuncture-Moxibustion in 1988.

This method was proposed in 1988 by an article entitled “The Project of the Standardization of AAPs” by the China Association of Acupuncture-Moxibustion in the Journal of Traditional Chinese Medicine [21] (Figure 10). The article put forward for the first time a new location method, in which AAPs were named mainly by the divided auricular subzones and by supplementing with the location method based on points, and also basically covered the anterior of the auricle. Advantages of this method are as follows. Firstly, it is based on the anatomy of the auricle, taking into account the complicated structure of the surface of the auricle. For AAPs at relatively flat area, the location method is based on subzones. For AAPs at tips of the different zones of the auricle or notches, the location method is based on points. It opens up a new thought of locating AAPs by different location methods according to the actual situation. Secondly, it is of great importance to create the location method which integrates auxiliary lines to locate AAPs according to the anatomical structures of the auricle, for example, at the ear lobe. The disadvantages are as follows. Firstly, a complete location principle was not formed. The application of the location method based on both points and subzones of the anterior of the auricle is not normative enough. For example, the location method based on points is still used for AAPs at the flat helix. Secondly, a considerable part of the anterior of auricle is still unmarked. Thirdly, there was no specification on how to locate AAPs on the posterior of the auricle.

### 3.7. The Location Method Based on Smaller Subzones of the Anterior of the Auricle

**Figure 11:**
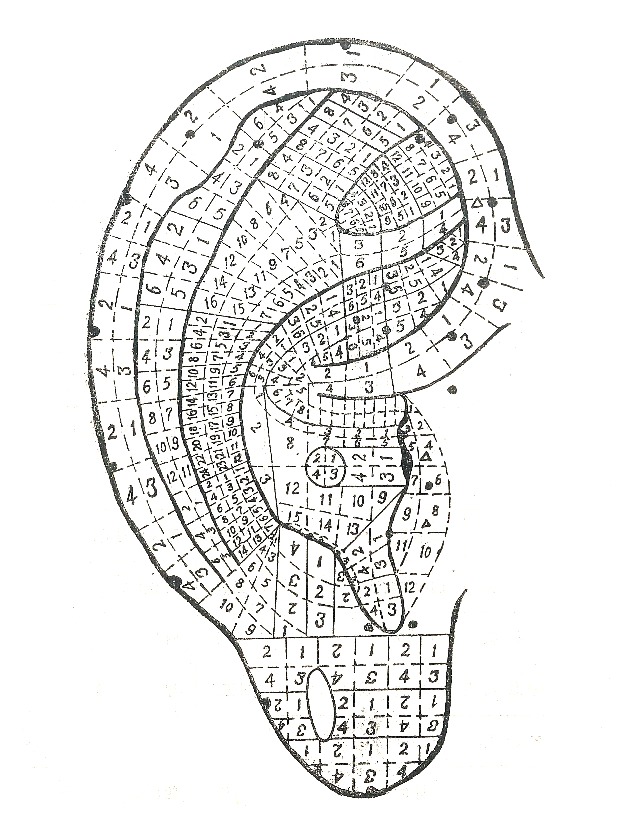
The location method based on smaller subzones of the anterior of the auricle in 1988.

**Figure 12:**
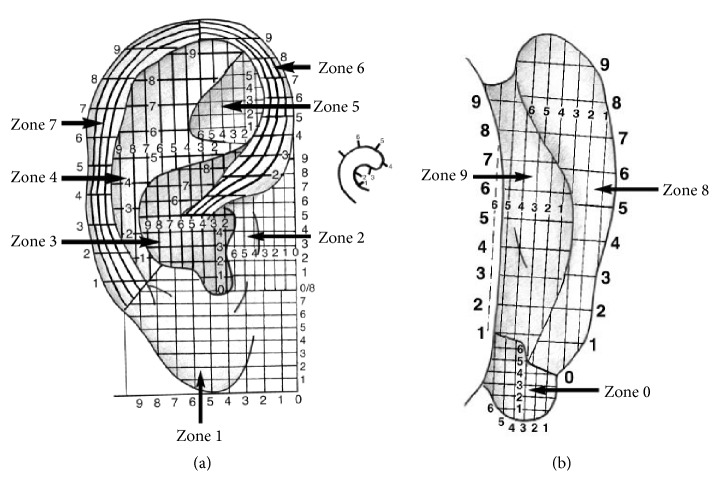
(a) Dr. Wojak's location method for the anterior of the auricle in 2010. (b) Dr. Wojak's location method for the posterior of the auricle in 2010.

The book,* Practical Auricular Acupuncture Therapy*, was published by Shanxi Science and Technology Press of China in 1988 which further divided the previous subzones into a number of even smaller subzones to improve the accuracy of the location of AAPs [22] (Figure 11). One similar location method [19], proposed by Dr. Winfred Wojak from Germany, divided the anterior auricle into 7 zones/areas and the posterior auricle into three zones/areas (Figures 12(a) and 12(b)). The anterior 7 zones included Zone 1, Zone 2, Zone 3, Zone 4, Zone 5, Zone 6, and Zone 7. The posterior 3 zones included Zone 8, Zone 9, and Zone 0. Each zone/area, except the helix, was divided into 9 vertical lines and 9 or 6 horizontal lines, which depended on the sizes of the zones/areas. For example, Zone 5 (the triangular fossa) and Zone 0 (the posterior of the lobe) were divided into 6 horizontal lines. The advantages of this method are as follows. Firstly, the location method inherits the objectivity and normativeness of the previous location method based on the anatomical structures of the auricle. Secondly, it considers the demand for an accurate description of the location of the point where the needle is inserted in the clinical practice of AAPs. It is helpful to choose the right point under the condition of the application of the location method based on subzones. Thirdly, this location method encompasses the idea, and subzones can be further divided into smaller subzones, which can be also further divided into even points. This idea unifies the location method based on points and the location method based on subzones according to the clinical demand. The shortcomings of this method are as follows. Firstly, the divided smaller subzones are even more complicated than the previous location method based on subzones, which brings difficulty in getting popular. Secondly, due to the complexity of the structures of the auricle, it is difficult to make further division of auricular zones according to the anatomical structures of the auricle. Therefore, this method does not become the mainstream location method for locating AAPs, though it is a helpful thought for the location of AAPs.

### 3.8. The Location Method Based on Subzones, Points, and Lines for the Anterior and Posterior of the Auricle

**Figure 13:**
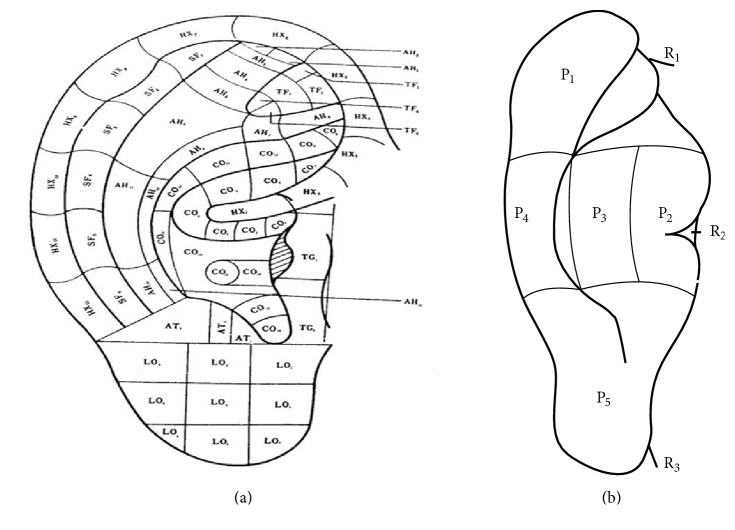
(a) The subzones of the anterior of the auricle [3, 4] and the international standard of auricular acupuncture points, issued by the World Federation of Acupuncture-Moxibustion Societies in 2013. (b) The subzones of the posterior of the auricle [3, 4] and the international standard of auricular acupuncture points, issued by the World Federation of Acupuncture-Moxibustion Societies in 2013.

**Figure 14:**
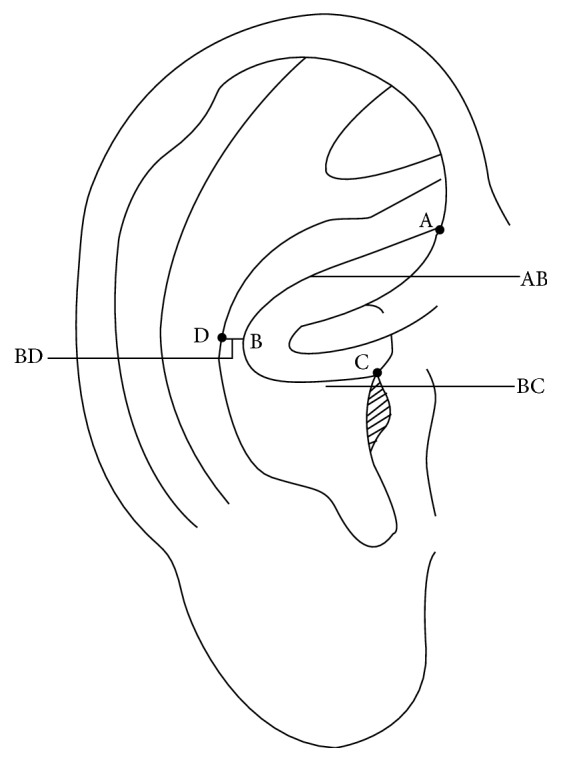
Marking points and lines in 1992.

**Figure 15:**
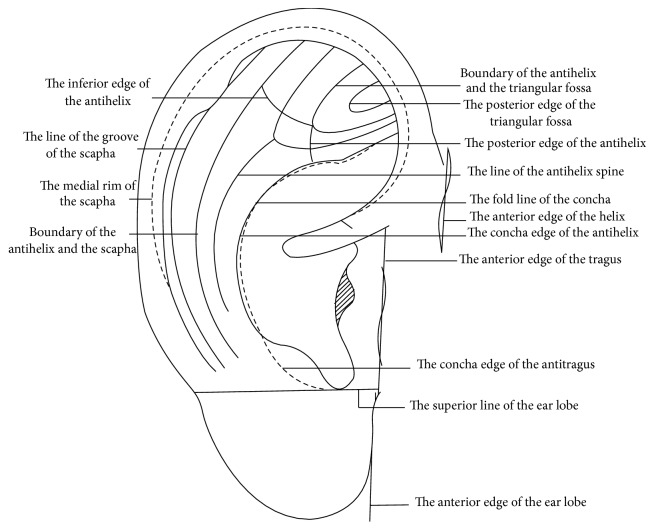
Marking lines in 1992.

After retaining the advantages and discarding the disadvantages of the above location methods, a new location method based on subzones, points, and lines for the anterior and posterior of the auricle was formed and applied in the clinical practice of AAPs. The Chinese National Standard, The Nomenclature and Location of AAPs, was issued in 1993. This standard systematically adopted this location method for the first time [3] (Figures 13(a) and 13(b)). The advantages of this new location method are as follows. Firstly, it keeps as many traditional locations of AAPs whose clinical therapeutic effects are proved as possible, which ensures the consistency of the nomenclature and location of auricular points. Secondly, it absorbs the advantages of all of the above location methods and also integrates the location method based on the natural auricular groove at the posterior of the auricle, making the location of AAPs more systematic. Thirdly, the idea of imaginary marking points and lines (Figures 14 and 15) proposed by the corresponding author, Professor Zhou [23], is adopted in this standard. Imaginary Point (A) is located at the medial edge of the helix at the junction between the middle and upper one-third of the line from the notch of helix crus and the inferior edge of the inferior antihelix crus. Imaginary Point (D) is located where a level line drawn from the end of the helix crus crosses the concha edge of the antihelix. Imaginary Point (B) is located at the junction of the middle and posterior one-third of the line extending from the end of the helix crus to Point (D). Imaginary Point (C) is located at the junction of the upper one-quarter and lower three-quarters of the posterior edge of the orifices of the external auditory meatus. Line (AB) is a curved line that extends from Point (A) to Point (B) and mirrors the concha edge of the antihelix. Line (BC) is a curved line extending from Point (B) to Point (C) that mirrors the inferior edge of the helix crus. In this way Z. Liqun makes it possible to describe accurately the locations of all AAPs according to the anatomical structures of the auricle and thus promotes the development of the standardization of localization of AAPs. Finally, it is helpful for the publication, research, teaching, and clinical application of AAPs. The limitation of this method is that there are still a small number of subzones unnamed, such as the anterior part of the helix and the two subzones at the anterior and posterior of the ear apex.

### 3.9. The Location Method Based on Subzones, Points, and Lines for Full Cover of the Anterior and Posterior of the Auricle

The method based on subzones according to the anatomical structures of the auricle, points, and lines for full cover of the anterior and posterior of the auricle was adopted by the new Chinese National Standard, Nomenclature and Locations of AAPs (GB/T13734-2008) [4] (Figures 13(a) and 13(b)) and also by the International Standard, Auricular acupuncture point (Figures 13(a) and 13(b)), issued by the World Federation of Acupuncture-Moxibustion Societies [5]. This location method marks the increasing maturity of the location of international standard of AAPs. Firstly, it accumulates the successful experience from the research work on international standardization of AAPs in the past 20 years and avoids the disadvantages of the above-mentioned location methods. Secondly, it names the two unnamed subzones in the former Chinese national standard in 1993 so that all subzones, points, and lines can be matched to a name, covering all areas of the auricle. However, this method still has some limitations. Firstly, there are some disagreements on the names of certain subzones or points between China and Europe. Secondly, some zones or subzones, especially the zones of the posterior of the auricle, are too big to insert the needle on the posterior of the auricle. The location method for the posterior of the auricle needs to be further improved.

## 4. Conclusion

The research on history, mechanism, and clinical application of auricular therapy has experienced a long process [24]. The nomenclature, location method, and consistency between name and location are three topics in the research field of international standardization of AAPs. Through analyzing the advantages and disadvantages of these nine representative location methods, we tried to offer a proper location method to locate AAPs.

The location method based on subzones according to the anatomical structures of the auricle, points, and lines for full cover of the anterior and posterior of the auricle is an appropriate method and is adopted by the WFAS international standard of AAPs. It is important to keep the right direction during developing an ISO international standard of auricular acupuncture points and to improve the research quality of international standardization for AAPs and of auricular diagnosis and treatment.
